# Orthostatic leg pain in neuropathic postural tachycardia syndrome: what does muscle excitability have to do with it?

**DOI:** 10.1007/s10286-021-00840-3

**Published:** 2021-11-12

**Authors:** Amy C. Arnold, Judith Navarro-Otano

**Affiliations:** 1. Department of Neural and Behavioral Sciences, Pennsylvania State University College of Medicine, Hershey, PA, USA; 2. Neurology Service, Hospital Clinic, Barcelona, Spain; 3. Institut d’Investigacions Biomèdiques August Pi i Sunyer (IDIBAPS), University of Barcelona, Barcelona, Spain

**Keywords:** orthostatic intolerance, neuropathic postural tachycardia syndrome, electromyography, muscle

Postural tachycardia syndrome (POTS) has emerged as one of the most common forms of chronic orthostatic intolerance and is currently estimated to affect millions of individuals globally. POTS is defined by the presence of excessive and sustained tachycardia upon standing with symptoms of orthostatic intolerance which are not explained by other causes, such as volume depletion, physical deconditioning, hyperventilation or drug effects. POTS contributes to considerable functional impairment and reduced quality of life [1,2]. This is a heterogenous disorder that consists of several subtypes often with overlapping clinical features. Of these subtypes, approximately half of patients are reported to have neuropathic POTS, which is characterized by variable degrees of peripheral sympathetic denervation primarily affecting the lower extremities [3]. Many patients exhibit acrocyanosis in the lower extremities and report lower limb muscle pain and weakness. While varying degrees of sympathetic denervation impairing reflex vasoconstriction during standing may contribute to excessive venous pooling and, thus, cause or aggravate these lower extremity symptoms, other factors might be involved [3,4]. Despite being a common complaint for these patients, few studies have investigated mechanisms contributing to orthostatic leg pain in POTS, with this phenomenon remaining understudied and poorly understood.

In this issue of *Clinical Autonomic Research*, Rodriguez and colleagues [5] examined muscle membrane properties in a small group of women with neuropathic POTS and with matched healthy controls. The tests were done in the supine position, following head-up tilt, and after recovery using a within-participants design. The function and properties of tibialis anterior muscle fiber membranes were measured *in vivo* by recording multi-fiber muscle velocity recovery cycles via concentric electromyography needles. The main finding of this study is that patients with neuropathic POTS have altered muscle excitability, with hyperpolarization while supine, progressive depolarization during head-up tilt, and a delayed recovery (Figure 1). The authors postulated that these muscle alterations reflect enhanced blood pooling in the lower extremities in these patients.

These findings may have particular importance for improving the understanding of the pathophysiology and management of neuropathic POTS. In this study, changes in electric muscle properties paralleled self-reported orthostatic leg pain levels. An ischemia-like process could explain the leg pain, discomfort, and weakness referred by patients with neuropathic POTS; however, it is worth noting that leg weakness was not specifically assessed in this study. Additionally, these findings complement a recent study showing accumulation of deoxygenated blood during head-up tilt and delayed muscle oxygenation during recovery in patients with POTS as measured by near-infrared spectroscopy muscle oximetry [6]. This prior study combined with the present findings may suggest that reductions in perfusion in the lower extremities as well as muscle excitability do not recover immediately in the supine position in POTS, which may correlate with the delayed leg pain recovery reported by these patients. The finding of muscle membrane depolarization during head-up tilt in POTS may suggest reduced potential for muscle activation, which could contribute to exercise intolerance, a common symptom in these patients. Conversely, we would expect that exercise therapy to increase hyperpolarization and reduce depolarization of muscle membranes, thus improving exercise tolerance in patients POTS, although this remains to be tested.

A potential area for consideration in the present study is the criteria used to classify patients as having neuropathic POTS. Currently, there are no specific diagnostic criteria for neuropathic POTS [3], and many of the tests used to assist in the diagnosis are not widely available in the general clinical setting. The definition of neuropathic POTS as a partial sympathetic denervation was initially suggested more than two decades ago, following studies that measured norepinephrine spillover in legs [7], a highly specialized technique not available in clinical practice. Other approaches to document peripheral autonomic nerve fiber impairment (such as quantitative sudomotor axon reflex testing, thermoregulatory sweat testing, and epidermal skin punch biopsy) also require special techniques that are often not easily accessible outside of tertiary care centers. Additionally, while patients in this study did not appear to have a hyperadrenergic phenotype, the impact of other subtypes that often have overlapping features, such as hypovolemic POTS, was not considered. Indeed, several of the patients diagnosed with neuropathic POTS in the present study reportedly had autoimmunity, which may be indicative of a different, underlying pathophysiology contributing to the patients’ symptoms.

The findings of this study raise intriguing questions. First, are these alterations in muscle membrane excitability exclusive to neuropathic POTS, applicable to POTS in general, or reflective of a subjective feeling of leg pain? This would require additional studies with control groups (i.e., women with leg pain with no POTS), patients with neuropathic POTS without leg pain, and patients with non-neuropathic POTS. Second, would these findings differ in patients with comorbid conditions that also influence venous pooling and muscle excitability? For example, some patients with orthostatic intolerance syndromes also have hypermobility, which may contribute to increased venous pooling [8]. While the participants in this study had no hypermobility, this remains an important question. Third, how do medications used to treat POTS symptoms impact posture-dependent muscle excitability measurements? Participants with neuropathic POTS were studied during continued medication use, including vasoactive drugs. While mimicking “real-world” conditions could be considered a strength of the study, in reality this is an important flaw, as it is conceivable that results may have been affected by these medications. This concern is heightened by the fact that these drugs were not allowed in the control group. So, is possible that we might be witnessing medication effects. Fourth, is the altered muscle excitability and symptoms of leg pain in POTS improved by exercise therapy or other interventions? A well-designed clinical trial will be required to answer this one. Finally, are similar changes in muscle membrane properties observed in patients with chronic and severe orthostatic venous pooling, such as the one caused by orthostatic hypotension?

Overall, the study by Rodriguez and colleagues is the first to examine for changes in muscle excitability in patients with POTS. These findings raise questions into the etiology and pathophysiology of leg pain in patients with POTS, specifically in patients with the neuropathic subtype, and may also have important implications for understanding exercise intolerance in these patients. Additional studies are needed to replicate these findings in a larger cohort of patients, who are not on any vasoactive drug, and who have leg pain due neuropathic and non-neuropathic POTS, and in patients with co-morbidities (e.g., hypermobility) other leg pain disorders that could potentially impact muscle function and properties.

## Figures and Tables

**Figure 1.**
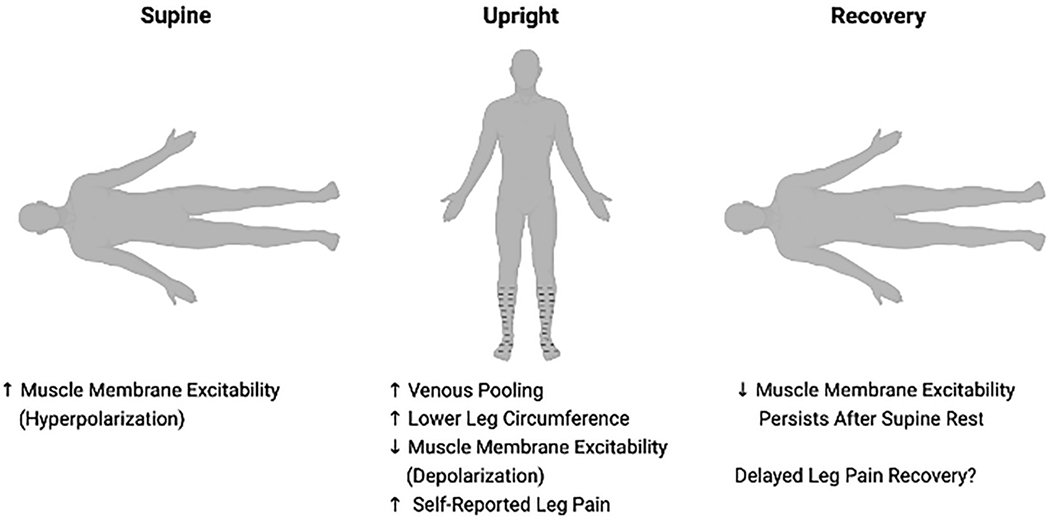
Overview of findings for altered muscle membrane excitability in patients with neuropathic POTS. Patients with neuropathic POTS exhibited altered muscle membrane excitability, with increased excitability (hyperpolarization) while supine, progressive decreased excitability (depolarization) during head-up tilt that was associated with increased lower leg circumference and self-reported leg pain. The decrease in muscle excitability persisted following supine recovery, which may, in part, explain the delayed leg pain recovery reported by these patients.
